# Father absence and trajectories of offspring mental health across adolescence and young adulthood: Findings from a UK-birth cohort

**DOI:** 10.1016/j.jad.2022.07.016

**Published:** 2022-10-01

**Authors:** Iryna Culpin, Hein Heuvelman, Dheeraj Rai, Rebecca M. Pearson, Carol Joinson, Jon Heron, Jonathan Evans, Alex S.F. Kwong

**Affiliations:** a Centre for Academic Mental Health, Population Health Sciences, Bristol Medical School, University of Bristol, Bristol, United Kingdom; b Leeds Institute of Health Sciences, School of Medicine, University of Leeds, United Kingdom; c NIHR Biomedical Research Centre, University of Bristol, Bristol, United Kingdom; d Avon and Wiltshire Partnership National Health Service (NHS) Trust, Bristol, UK; e Centre for Academic Child Health, Population Health Sciences, Bristol Medical School, University of Bristol, Bristol, United Kingdom; f MRC Integrative Epidemiology Unit, University of Bristol, Bristol, United Kingdom; g Division of Psychiatry, Centre for Clinical Brain Sciences, University of Edinburgh, Edinburgh, United Kingdom

**Keywords:** ALSPAC, Biological father absence, Offspring depression, Trajectories of depressive symptoms, Population-based study

## Abstract

**Background:**

High prevalence of parental separation and resulting biological father absence raises important questions regarding its impact on offspring mental health across the life course. We specifically examined whether these relationships vary by sex and the timing of exposure to father absence (early or middle childhood).

**Methods:**

This study is based on up to 8409 children from the Avon Longitudinal Study of Parents and Children (ALSPAC). Participants provided self-reports of depression (Clinical Interview Schedule-Revised) at age 24 years and depressive symptoms (Short Mood and Feelings Questionnaire) between the ages of 10 and 24 years. Biological father absence in childhood was assessed through maternal questionnaires at regular intervals from birth to 10 years. We estimated the association between biological father absence and trajectories of depressive symptoms using multilevel growth-curve modelling.

**Results:**

Early but not middle childhood father absence was strongly associated with increased odds of offspring depression and greater depressive symptoms at age 24 years. Early childhood father absence was associated with higher trajectories of depressive symptoms during adolescence and early adulthood compared with father presence. Differences in the level of depressive symptoms between middle childhood father absent and father present groups narrowed into adulthood.

**Limitations:**

This study could be biased by attrition and residual confounding.

**Conclusions:**

We found evidence that father absence in childhood is persistently associated with offspring depression in adolescence and early adulthood. This relationship varies by sex and timing of father's departure, with early childhood father absence emerging as the strongest risk factor for adverse offspring mental health trajectories Further research is needed to identify mechanisms that could inform preventative interventions to reduce the risk of depression in children who experience father absence.

## Highlights

• Father absence during early childhood is associated with greater levels of depression in early adulthood.
• Early childhood father absence is associated with more severe depression trajectories across adolescence and early adulthood.
• Effects are strongest for females with absent fathers in early childhood.

## 1 Introduction

Recent evidence suggests that a substantial proportion of marriages (42 %; ONS, 2013) and cohabiting relationships (27 %; Crawford et al., 2011) in the UK are likely to end in divorce and separation, with around half of these divorces and separations expected to occur in the first 10 years. It is estimated that the majority of children reside with the mother (74.3 % in UK) following divorce and/or parental separation (Kalmijn, 2015; ONS, 2013). The high prevalence of parental divorce/separation and the ensuing absence of the biological father from the household raises important questions regarding its impact on offspring mental health at different developmental time points and across the life course. Ample longitudinal evidence emphasises the adverse impact of parental divorce, separation and biological father absence on child and adolescent mental health, whilst accounting for sociodemographic and familial factors (Amato, 2001; Amato and Keith, 1991; Culpin et al., 2013; McLanahan et al., 2013). However, it remains unclear whether father absence during childhood is associated with increased risk of mental health difficulties in adulthood (Amato and Keith, 1991).

The association between biological father absence and mental health may depend on the child's sex and developmental stage when the father left (Oldehinkel et al., 2008; Sigle-Rushton and McLanahan, 2004). It has been suggested that father absence that occurs during early childhood (birth to 5 years) is associated with higher risk of depression in adolescence than paternal absence later in childhood (5–10 years; Culpin et al., 2013; Ermisch and Francesconi, 2001; Rudolph, 2002). However, these findings are inconsistent, and, importantly, it remains unknown whether adverse effects of father absence timing persist into early adulthood (McLanahan et al., 2013). The evidence with regard to sex differences in the association between father absence and offspring depression is similarly inconclusive (McLanahan et al., 2013), with some studies reporting stronger effects for females (Culpin et al., 2013; Oldehinkel et al., 2008), whilst other studies suggest that males are worse affected (Morrison and Cherlin, 1995; Størksen et al., 2005).

Offspring mental health is a dynamic developmental process that unfolds between individuals and their context (Sameroff, 2000). Thus, it is important to assess individual variation in changes in mental health over time following childhood father absence. Explicit longitudinal modelling of mental health trajectories can assess individual variation across development and estimate changes in mental health over time across childhood into adulthood (Herle et al., 2020). In addition, it enables estimation of age-specific effects (early- versus middle-childhood), the persistence of effects over time, and interaction with other characteristics such as sex (Ge et al., 2006).

Several studies have examined trajectories of offspring mental health following parental separation. Ge et al. (2006) found that children from divorced families had higher trajectories of depressive symptoms across adolescence compared to children from nondivorced families, and that this effect was stronger for females. Cherlin et al. (1998) found a pronounced adverse effect of parental divorce in adolescence on offspring trajectories of depressive symptoms between ages 7 and 33 years suggesting long lasting effects well into adulthood. Similarly, Costello et al. (2008) reported that children from separated families were at higher risk of belonging to a higher trajectory of depressive symptoms from ages 12 to 25 years. However, these findings are limited by the selective and relatively small sample size (Ge et al., 2006), use of depression measures not validated in adolescence (Costello et al., 2008), and relatively large gaps between assessments (Cherlin et al., 1998). Crucially, none of the studies have explicitly examined the association between biological father absence and depressive symptom trajectories, nor have they looked at the timing of father absence, or sex differences in this association.

In the current study we address this gap in literature using data from a large UK-based birth cohort, the Avon Longitudinal Study of Parents and Children (ALSPAC), to estimate the association between biological father absence in childhood and population trajectories of depressive symptoms between ages 10 and 24 years using multilevel growth-curve modelling. We used longitudinal data with frequent (bi-yearly) assessments of depressive symptoms throughout early childhood, adolescence and early adulthood using well-validated measures of child and adolescent psychopathology and adjusting for a range of confounding factors preceding father departure, including marital conflict.

Our specific research questions were:

1. Are offspring whose biological fathers were absent in early (birth to 5 years) and later (5–10 years) childhood at increased risk of depression at age 24 years, and does this risk vary by sex and timing of father absence?
2. Do trajectories of depressive symptoms between the ages 10 and 24 years for children whose biological fathers were present or absent in early (birth to 5 years) and later (5–10 years) childhood differ?

## 2 Methods

### 2.1 Study cohort

The sample comprised participants from the Avon Longitudinal Study of Parents and Children (ALSPAC). During Phase I enrolment, 14,541 pregnant mothers residing in the former Avon Health Authority in the South-West of England with expected dates of delivery between 1 April 1991 and 31 December 1992 were recruited. The total enrolled ALSPAC sample size is 15,454 pregnancies, of which 14,901 were alive at 1 year of age. Ethical approval for the study was obtained from the ALSPAC Ethics and Law Committee and the Local Research Ethics Committees. The study website (www.bristol.ac.uk/alspac/) contains details of all the data that is available through a fully searchable data dictionary and variable search tool: http://www.bris.ac.uk/alspac/researchers/our-data. Further details on the cohort profile, representativeness and phases of recruitment are described in three cohort-profile papers (Boyd et al., 2013; Fraser et al., 2012; Northstone et al., 2019). Study data gathered from participants at 22 years and onwards were collected and managed using REDCap electronic data capture tools hosted at the University of Bristol (Harris et al., 2009).

### 2.2 Measures

#### 2.2.1 Exposure: biological father absence in childhood

Absence of the biological father was assessed through maternal self-reported questionnaires at regular intervals since the birth of the study child (full details in Methods S1, Supplementary). The questions enquired whether the present live-in father figure is the natural father of the study child, and if not, how old the study child was when the biological father stopped living with the family. Data on biological father absence were divided into two distinct age periods to capture father absence during early (birth-5 years) and later (5–10 years) childhood. The majority of fathers who were absent left before the child's first birthday, with over 20 % of fathers absent from the birth of the study child. The proportion of absent fathers declined steadily over the subsequent years, with only 3.04 % of mothers reporting biological father absence from the household between the ages 9 and 10 years.

#### 2.2.2 Outcome: offspring depression and depressive symptoms

Offspring depression was assessed using the computerised version of the Clinical Interview Schedule-Revised (CIS-R; Lewis et al., 1992), a fully structured psychiatric interview widely used in the community samples (Thapar and McGuffin, 1998). It was administered at the research clinic at age 24 years to identify individuals with an ICD-10 diagnosis of depression (versus no diagnosis).

Offspring depressive symptoms were assessed using the Short Mood and Feelings Questionnaire (SMFQ; Angold et al., 1995) on nine occasions between ages 10 and 24 years (full details in Methods S1, Supplementary). Scores for the individual items were summed up to produce a summary score (range 0–26). A binary variable was also derived with a cut-off point of ≥10, shown to have high sensitivity and specificity for diagnosed depression (Turner et al., 2014), to identify individuals with depressive symptoms at age 24 years (versus no depressive symptoms). The SMFQ has been validated in late adolescence (Patton et al., 1999) and correlates highly with the Diagnostic Interview Schedule for Children (Shaffer et al., 2000). Details on the use of SMFQ in longitudinal models have been reported previously (Kwong et al., 2019).

### 2.3 Potential confounders

Disadvantaged socioeconomic status and marital conflict are strong risk factors for biological father absence and offspring depression (McLanahan et al., 2013; Sigle-Rushton and McLanahan, 2004). Analyses were adjusted for a range of potential confounding factors collected prospectively from maternal antenatal questionnaires, including: financial problems (occurrence of major financial problems since pregnancy versus none); maternal educational attainment (three-level scale: minimal education or none, compulsory secondary level (up to age 16 years; O-Level), A Levels/university degree), parental occupational class (professional/managerial versus manual), homeownership status (three-level scale: owned/mortgaged, privately-rented, council-rented), maternal depression (18 weeks gestation) assessed using the Edinburgh Postnatal Depression Scale (EPDS; sum-score; Cox et al., 1987), and a measure of parental conflict with higher scores indicating higher levels of inter-parental conflict.

### 2.4 Statistical analyses

Full details of the statistical analyses, including trajectories, are presented in Methods S1, Supplementary. In summary, we examined characteristics of the sample (Table S1) and prevalence of depression diagnosis and depressive symptoms by the presence or absence of the biological father (Table S2, Supplementary). We used binary logistic regression (*logit* command) to examine the association between father absence occurring during different periods in childhood (early: birth-5 years and later: 5–10 years) and ICD-10 depression diagnosis and depressive symptoms at age 24 years. First, we compared children exposed to father absence from birth-5 years (*n* = 1943) with all children whose fathers were present between birth-10 years (*n* = 9003). We included in this group the 726 children whose fathers were absent during the period 5–10 years because, by definition, they were not absent during the first 5 years of life. Second, we compared children exposed to father absence during the period 5–10 years (*n* = 726) with those whose fathers were present throughout the period 0–10 years (*n* = 8277). We then tested for an interaction between father absence and sex using the likelihood ratio test in the full sample to maximise power to detect interaction effect. We estimated models unadjusted and adjusted for the confounding factors.

Finally, we examined trajectories of depressive symptoms (continuous SMFQ scores) between the ages of 10 and 24 years among those whose fathers were absent/present and then again separately for males and females using multilevel growth-curve models with random intercepts and random slopes to examine how depressive symptoms changed across development for each father absence model (details in Methods S1, Supplementary). We ran both adjusted and unadjusted models to match the depression diagnosis analyses. We included linear, quadratic, cubic and quartic polynomial age terms in the models to accommodate the non-linearity of the trajectories as per previous research (Kwong et al., 2021). We interacted the age terms with the main effect of early and later childhood father absence in two separate models, followed by age terms interacted with sex across the two separate models to create trajectories for each risk group (Results S1, Supplementary). To aid interpretation of the non-linear growth curves, we calculated the predicted mean scores for each trajectory at ages 12, 16, 20 and 24 years, followed by calculating the predicted mean difference at the varying ages between trajectories. To correct for multiple comparison testing with the predicted mean difference analysis, *p*-values were corrected using the false discovery rate. All analyses were conducted using Stata v.15/MP (StataCorp., USA), including trajectories estimated using the *runmlwin* command (Leckie and Charlton, 2013).

### 2.5 Missing data: multiple imputation, full information maximum likelihood and inverse probability weighted analysis

Characteristics of the study sample by completeness of the data are presented in Results S1 and Table S3, Supplementary. We conducted sensitivity analyses to examine the impact of missing data on our findings. Full description of the imputation methods, including trajectories (full information maximum likelihood and inverse probability analysis to handle missing data), are presented in Method S1, Supplementary.

## 3 Results

### 3.1 Study sample derivation

**Fig. 1:**
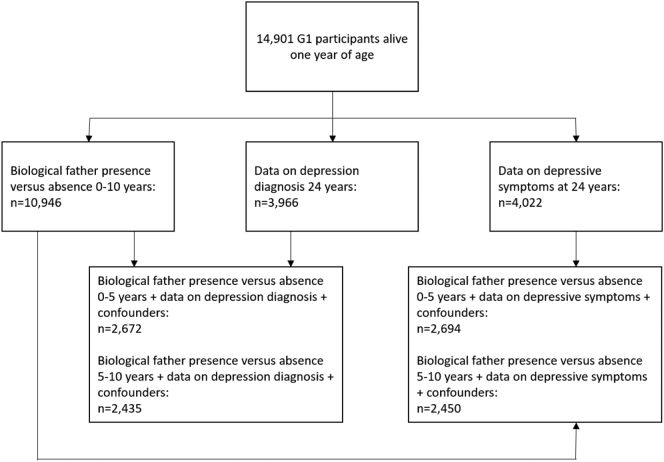
Study sample.

Fig. 1 represents sample sizes with complete data on exposure, outcome and confounding factors to examine the effects of biological father absence on offspring depression diagnosis and depressive symptoms at age 24 years (full details in Results S1, Supplementary). The sample sizes for those with data on exposure, confounding factors and at least one measurement of depressive symptoms to establish trajectories was 6020 (early father absence birth-5 years) and 5352 (later father absence 5–10 years). The majority of fathers were absent during the first five years of child's life (17,7 %), with the proportion of absent fathers declining between ages 5–10 years (6.6 %).

### 3.2 Association between father absence in childhood and depression (diagnosis and symptoms) at age 24 years

Distribution of depression, socioeconomic and familial characteristics in father-present and father-absent samples is presented in Results S1 and Tables S1-S2. Of 3966 young adults with data on depression diagnosis and 4022 with data on depressive symptoms at age 24 years, 432 (10.9 %, 95%CI 0.09, 0.12) met the criteria for depression, and 994 (24.7 %, 95%CI 0.23, 0.26) reported depressive mood. First, we estimated the main effect of father absence and the interaction between father absence and sex in the full sample to maximise power to detect possible interactions. There was evidence of the main effect of father absence in early (OR: 1.97, 95%CI 1.49, 2.60, *p* ≤ 0.001; Table 1), but not later childhood (OR: 1.13, 95%CI 0.73, 1.76, *p* = 0.585; Table 1), on depression diagnosis at age 24 years. There was no evidence for an interaction between sex and father absence in early childhood, indicating that the association between early childhood father absence and diagnosis of depression at age 24 years is similar for boys and girls (Table 1). There was evidence for a main effect of father absence in early (OR: 1.54, 95%CI 1.21, 1.93, p ≤ 0.001) and later (OR: 1.43, 95 % CI 1.10, 1.95, *p* = 0.020) childhood on depressive symptoms at age 24 years (Table 1). There was weak evidence for an interaction between sex and father absence in later, but not early childhood, on depressive symptoms at age 24 years (Table 1).

**Table 1:** Odds ratios for [95 % Cis] for the association between father absence during different periods in childhood and binary indicators of depression diagnosis and depressive symptoms at 24 years stratifying by sex in the entire sample.

Risk factor: Timing of father absence	Depression diagnosis (CIS-R) at 24 years (Reference = No depression diagnosis)			
	n	Main effect of father absence^a^	Main effect of father absence+interaction term for sex^b^	Test for interaction^c^
		OR [95 % CI], p	OR [95 % CI], p	Chi2, p
Early father absence (birth-5 years) (Reference = Father present)	3565	1.97 [1.49, 2.60], *p* ≤ 0.001	2.52 [1.50, 4.23], p ≤ 0.001	1.30, *p* = 0.255
Gender (Reference = Male)		1.94 [1.51, 2.45], p ≤ 0.001	2.05 [1.57, 2.69], p ≤ 0.001	
Father absence x sex		–	0.70 [0.38, 1.29], *p* = 0.249	
Later father absence (5–10 years) (Reference = Father present)	3151	1.13 [0.73, 1.76], p = 0.585	0.60 [0.18, 1.95], *p* = 0.398	1.49, *p* = 0.222
Gender (Reference = Male)		2.05 [1.57, 2.69], p ≤ 0.001	1.97 [1.49, 2.59], p ≤ 0.001	
Father absence x sex		–	2.10 [0.59, 7.49], p = 0.255	
	Depressive symptoms (SMFQ) at 24 years (Reference = No depressive symptoms)			
Early father absence (birth-5 years) (Reference = Father present)	3633	1.54 [1.24, 1.93], p ≤ 0.001	1.84 [1.22, 2.78], p = 0.003	0.96, *p* = 0.328
Gender (Reference = Male)		1.58 [1.34, 1.87], p ≤ 0.001	1.63 [1.37, 1.96], p ≤ 0.001	
Father absence x sex		–	0.78 [0.48, 1.27], *p* = 0.325	
Later father absence (5–10 years) (Reference = Father present)	3207	1.43 [1.10, 1.95], p = 0.020	0.85 [0.44, 1.64], *p* = 0.621	3.70, *p* = 0.054
Gender (Reference = Male)		1.63 [1.36, 1.95], p ≤ 0.001	1.55 [1.29, 1.87], p ≤ 0.001	
Father absence x sex		–	2.03 [1.00, 4,29], *p* = 0.064	

OR: odds ratio. CIS-R: Clinical Interview Schedule-Revised. SMFQ: Short Mood and Feelings Questionnaire.

a Unadjusted full sample: individuals with data on the different levels of father absence and later depression diagnosis/depressive symptoms.

b The interaction term quantifying the interaction between the different father absence levels by sex.

c Test for interaction is the likelihood ratio test comparing models with and without the interaction term, quantified by a Chi2 test and related *p* value.

Second, we estimated unadjusted and adjusted models to substantiate our findings with regard to the main effects of early and later childhood father absence on depression diagnosis and depressive symptoms at age 24 years whilst accounting for a range of confounders. Consistent with the analyses utilising the whole sample, there was strong evidence in the unadjusted model for a main effect of father absence in early childhood on depression (OR: 2.00, 95%CI 1.39, 2.89, p ≤ 0.001; Table 2) and depressive symptoms (OR: 1.86, 95%CI 1.40, 2.46, p ≤ 0.001; Table 2) at age 24 years. Although moderately attenuated, this association was independent of socioeconomic, maternal and familial confounders (depression: OR: 1.58, 95%CI 1.07, 2.32, *p* = 0.021; depressive symptoms: OR: 1.52, 95%CI 1.13, 2.03, *p* = 0.006; Table 2). In contrast, there was no evidence for a main effect of father absence later in childhood on depression or depressive symptoms at age 24 years in the unadjusted (depression: OR: 1.13, 95%CI 0.67, 1.90, *p* = 0.650; depressive symptoms: OR: 1.17, 95%CI 0.80, 1.72, *p* = 0.424; Table 2) or adjusted models (depression: OR: 0.97, 95%CI 0.57, 1.65, *p* = 0.906; depressive symptoms: OR: 1.04, 95%CI 0.70, 1.54, *p* = 0.842; Table 2). The results from the analyses with imputed data supported our findings and led to the same overarching conclusions (Results S1, Tables S4-S5, Supplementary).

**Table 2:** Odds ratios [95 % CI] for the main effect of father absence during different periods in childhood on binary indicators of depression diagnosis and depressive symptoms at 24 years.

Risk factor: timing of father absence	n	Depression diagnosis (CIS-R) at 24 years (Reference = no depression diagnosis)		n	Depressive symptoms (SMFQ) at 24 years (Reference = no depressive symptoms)	
Father absence (Reference = Father present)		Unadjusted OR [95 % CI], p	Adjusted^a^ OR [95 % CI], p		Unadjusted OR [95 % CI], p	Adjusted^a^ OR [95 % CI], p
Early father absence (birth-5 years)	2672	2.00 [1.39, 2.89], p ≤ 0.001	1.58 [1.07, 2.32], p = 0.021	2694	1.86 [1.40, 2.46], p ≤ 0.001	1.52 [1.13, 2.03], *p* = 0.006
Later father absence (5–10 years)	2435	1.13 [0.67, 1.90], p = 0.650	0.97 [0.57, 1.65] p = 0.906	2450	1.17 [0.80, 1.72], p = 0.424	1.04 [1.70, 1.54], p = 0.842

*Note*: ^a^Adjusted for antenatal indicators of socioeconomic (parental social class, financial difficulties, maternal educational attainment, homeownership), maternal (depression) and familial (parental conflict) characteristics. OR: Odds ratio. CIS-R: Clinical Interview Schedule-Revised. SMFQ: Short Mood and Feelings Questionnaire.

### 3.3 Association between father absence in childhood and trajectories of depressive symptoms from childhood to early adulthood

**Fig. 2:**
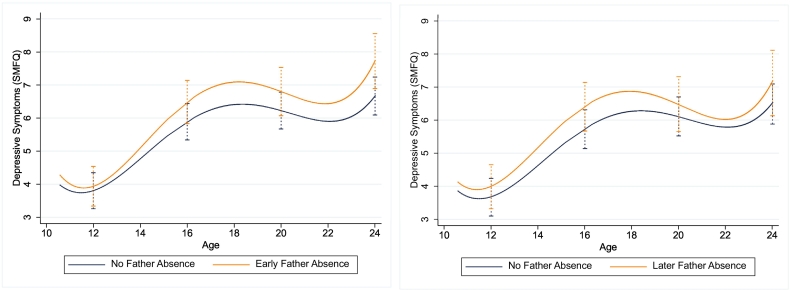
Main effect of father absence early in childhood (birth-5 years; left) and father absence in middle childhood (5–10 years; right) on predicted trajectories of depressive symptoms across childhood, adolescence and young adulthood.

As non-linear trajectories are difficult to interpret, the predicted mean differences between different groups at different ages are presented in Table 3, with regression coefficients from the models presented in Tables S6-S9 and Tables S10-S13, Supplementary. The predicted scores for each group at different ages are presented in Table S14, Supplementary. In the adjusted model, the overall pattern of depressive symptoms trajectories increased throughout later childhood and adolescence up to age 18 years, followed by a decrease around age 20 before rising again around age 22 years. Children exposed to father absence in early childhood, compared with presence, had similar trajectories around age 12 years, but began to have higher trajectories of depressive symptoms from around age of 14 (Fig. 2). This increase in trajectories was indexed by father absence in early childhood having a greater mean difference in depressive symptoms scores from the age of 16 years (Beta^diff^ = 0.60, 95%CI 0.17, 1.02, *p* = 0.012; Table 4), with the greatest difference observed at age 24 (Beta^diff^ = 1.06, 95%CI 0.37, 1.74, *p* = 0.003; Table 4). Father absence later in childhood showed a similar pattern up to the age of 16 years with evidence of a mean difference observed at this age (Beta^diff^ = 0.68, 95%CI 0.17, 1.19, *p* = 0.008; Table 4). However, in contrast to father absence in early childhood, father absence later in childhood was not associated with any mean differences at age 20 or 24 years respectively as the gap between father absence later in childhood and father presence during this period trajectories narrowed into early adulthood. Results did not differ substantially with the inverse probability weighted analysis (Table S15-S16, Supplementary).

**Table 3:** Predicted mean difference in depressive symptoms scores [95 % CI] at various ages for the main effect of father absence during different periods in childhood on trajectories of depressive symptoms.

	Predicted Mean Differences in Depressive Symptoms Scores (SMFQ) ^a^			
*Early Father Absence (EFA; n* = 6020)	Age 12	Age 16	Age 20	Age 24
No EFA vs Yes EFA	0.13 (−0.19, 0.45), *p* = 0.426	0.60 (0.17, 1.02), p = 0.012	0.57 (0.04, 1.11), *p* = 0.048	1.06 (0.37, 1.75), p = 0.003
*Later Father Absence (LFA; n* = 5352)				
No LFA vs Yes LFA	0.31 (−0.07, 0.69), *p* = 0.190	0.68 (0.17, 1.19), p = 0.008	0.37 (−0.27, 1.01), *p* = 0.254	0.63 (−0.21, 1.48), *p* = 0.143

*Note*: ^a^ Adjusted for antenatal indicators of socioeconomic (parental social class, financial difficulties, maternal educational attainment, homeownership), maternal (depression) and familial (parental conflict) characteristics. *P*-values are adjusted for false discovery rate (FDR). SMFQ: Short Mood and Feelings Questionnaire. EFA: Early Father Absence. LFA: Later Father Absence.

**Table 4:** Predicted mean difference in depressive symptoms scores [95 % CI] at various ages for the main effect of father absence during different periods in childhood on trajectories of depressive symptoms stratifying by sex in the entire sample.

	Predicted mean differences in depressive symptoms scores (SMFQ)^a^			
*Early Father Absence (EFA; n* *=* *6020)*	Age 12	Age 16	Age 20	Age 24
Male No EFA vs Female No EFA	0.40 (0.19, 0.60), *p* = 0.0002	2.06 (1.80, 2.32), *p* = 1.6 × 10^−15^	1.56 (1.24, 1.89), *p* = 1.6 × 10^−15^	1.37 (0.93, 1.80), *p* = 2.7 × 10^−09^
Male No EFA vs Male Yes EFA	0.01 (−0.47, 0.45), *p* = 0.974	0.52 (−0.11, 1.15), p = 0.14	0.25 (−0.61, 1.11), *p* = 0.624	2.11 (0.95, 3.27), *p* = 0.0009
Male No EFA vs Female Yes EFA	0.65 (0.21, 1.10), *p* = 0.007	2.68 (2.13, 3.24), p = 1.6 × 10^−15^	2.25 (1.56, 2.94), *p* = 1.1 × 10^−09^	1.87 (0.99, 2.74), *p* = 0.00009
Female No EFA vs Male Yes EFA	0.40 (−0.06, 0.87), *p* = 0.12	1.54 (0.91, 2.16), *p* = 4.8 × 10^−06^	1.32 (0.47, 2.17), *p* = 0.004	0.74 (0.40, 1.88), *p* = 0.253
Female No EFA vs Female Yes EFA	0.26 (−0.19, 0.70), *p* = 0.294	0.63 (0.07, 1.18), *p* = 0.044	0.69 (0.01, 1.36), *p* = 0.072	0.50 (−0.35, 1.36) p = 0.294
Male Yes EFA vs Female Yes EFA	0.66 (0.06, 1.26), *p* = 0.049	2.16 (1.37, 2.95) *p* = 3.3 × 10^−07^	2.00 (0.96, 3.05), p = 0.0002	0.24 (−1.13, 1.61), *p* = 0.762
*Later Father Absence (LFA; n* *=* *5352)*				
Male No LFA vs Female No LFA	0.38 (0.18, 0.59), *p* = 0.0006	1.94 (1.67, 2.20), p = 1.6 × 10^−15^	1.45 (1.12, 1.79), *p* = 1.6 × 10^−15^	1.28 (0.84, 1.72), *p* = 3.6 × 10^−08^
Male No LFA vs Male Yes LFA	0.28 (−0.27, 0.94), *p* = 0.362	0.34 (−0.44, 1.11), *p* = 0.429	0.67 (−0.38, 1.72), p = 0.253	0.06 (−1.43, 1.54), p = 0.939
Male No LFA vs Female Yes LFA	0.80 (0.27, 1.33), p = 0.006	3.20 (2.55, 3.85), p = 1.6 × 10^−15^	2.37 (1.56, 3.18), *p* = 3.7 × 10^−08^	2.34 (1.26, 3.41), *p* = 0.00005
Female No LFA vs Male Yes LFA	0.10 (−0.46, 0.65), *p* = 0.759	2.28 (1.50, 3.05), *p* = 3.5 × 10^−08^	2.12 (1.08, 3.16), *p* = 0.0001	1.34 (−0.14, 2.81), *p* = 0.105
Female No LFA vs Female Yes LFA	0.42 (−0.11, 0.95), *p* = 0.162	1.26 (0.61, 1.91), p = 0.0002	0.92 (0.12, 1.72), p = 0.038	1.06 (0.00, 2.12), p = 0.076
Male Yes LFA vs Female Yes LFA	0.52 (−0.22, 1.25), p = 0.215	3.53 (2.56, 4.51), *p* = 6.2 × 10^−12^	3.04 (1.77, 4.32), *p* = 8.7 × 10^−06^	2.39 (0.62, 4.17), *p* = 0.013

*Note*: ^a^Adjusted for antenatal indicators of socioeconomic (parental social class, financial difficulties, maternal educational attainment, homeownership), maternal (depression) and familial (parental conflict) characteristics. P-values are adjusted for false discovery rate (FDR). SMFQ: Short Mood and Feelings Questionnaire. EFA: Early Father Absence. LFA: Later Father Absence.

**Fig. 3:**
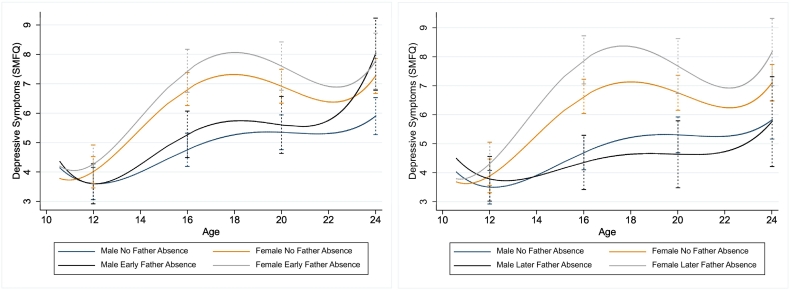
Main effect of early father absence (left; birth-5 years) and later father absence in middle childhood (right; 5–10 years) on predicted trajectories of depressive symptoms across childhood, adolescence and young adulthood, stratified by sex.

There was clear evidence that females had higher trajectories of depressive symptoms compared to males across adolescence and young adulthood (Fig. 3). Females exposed to father absence in early and middle childhood had the highest trajectories, with males whose fathers were present across childhood displaying the lowest trajectories. Males whose fathers were absent in early childhood had similar trajectories to those whose fathers were present until the age 24 years, followed by a steep rise in depressive symptoms with a greater mean difference in depressive symptoms scores at age 24 years (Beta^diff^ = 2.11, 95%CI 0.95, 3.27, *p* = 0.009; Table 4). Compared with females whose fathers were present, those whose fathers were absent in early childhood had higher depressive symptoms trajectories throughout adolescence, but this difference narrowed by age 24 (Beta^diff^ = 0.50, 95%CI -0.35, 1.36, *p* = 0.294; Table 4). Compared with males, females who were exposed to father absence in early childhood had higher depressive symptom trajectories across late childhood and adolescence, but the sharp rise in depressive symptoms in adulthood for males exposed to early father absence resulted in trajectories that were non-discriminant at age 24 years (Beta^diff^ = 0.24, 95%CI -1.13, 1.61, *p* = 0.762; Table 4).

There was no steep rise in depression trajectories in adulthood observed for males whose father was absent later in childhood, with little difference in depressive symptoms scores at age 24 years between father present males (Beta^diff^ = 0.06, 95%CI -1.43, 1.54, *p* = 0.939; Table 4). In contrast, males with later father absence had the lowest trajectories of all, although these were non-discriminant from males whose fathers were present. There was strong evidence that females exposed to father absence later in childhood had higher depression trajectories than their unexposed female counterparts at age 16 (Beta^diff^ = 1.26, 95%CI 0.61, 1.91, *p* = 0.002) and 20 years (Beta^diff^ = 0.92, 95%CI 0.12, 1.72, *p* = 0.038), though this difference had narrowed somewhat by age 24 years (Beta^diff^ = 1.06, 95%CI 0.00, 2.12, *p* = 0.076; Table 4). Similarly, to early father absence model, females whose fathers were absent later in childhood had higher depressive symptoms trajectories compared to males whose fathers left later in childhood across all ages except age 12 years (Beta^diff^ = 0.52, 95%CI -0.22, 1.25, *p* = 0.215; Table 4).

## 4 Discussion

### 4.1 Main findings

In this large population-based study we found that early but not later childhood father absence was associated with increased odds of offspring depression at age 24. We found no evidence for a sex difference in this relationship. Father absence in both early and later childhood was associated with increased odds of depressive symptoms at age 24 years, with some evidence of this relationship being stronger for females than males. Consistently, father absence in early childhood was strongly associated with increased odds of offspring depression and depressive symptoms at age 24 years in the unadjusted and fully adjusted models. Although moderately attenuated, these associations were independent of socioeconomic, maternal and familial confounding factors. In contrast, there was no evidence to suggest that father absence later in childhood was associated with increased odds of either offspring depression or depressive symptoms at age 24 years in the unadjusted and fully adjusted models.

Modelling developmental trajectories of depressive symptoms enabled us to track the effects of biological father absence during different periods in childhood on offspring mental health over time, providing insights into whether these effects persist or diminish with age. The overall pattern of depressive symptoms was consistent with that observed in previous studies, with initially low levels of depressive symptoms in late childhood increasing through adolescence up to age 18, followed by a decline before rising again at the age of 22 (Kwong, 2019). There was clear evidence that females, on average, had much worse trajectories compared to father absent and father present males across adolescence and young adulthood, with males whose fathers were present throughout childhood displaying the lowest trajectories across development. These findings are consistent with previous research suggesting that females are more likely than males to have increasing trajectories of depressive symptoms from childhood through late adolescence (Dekker et al., 2007; Lewis et al., 2020) and early adulthood (Adkins et al., 2009).

Father absence in early childhood was associated with higher depressive symptom trajectories across adolescence with the greatest difference in mean depressive symptoms scores between father absent and present groups observed at age 24 years. This suggests that there is a persistent association between early father absence and depressive symptoms in late adolescence and young adulthood. Offspring whose fathers were absent later in childhood had similar trajectories until mid-adolescence. However, in contrast to the effects of early father absence, the gap between offspring whose father were absent and present later in childhood tended to narrow into early adulthood, suggesting that there may be differential mechanisms underlying timing effects of early father absence. In line with the family stress model (Conger et al., 1990), early childhood adverse experiences, including parental separation and divorce, may trigger acute and chronic economic hardship (Conger et al., 2010), parental conflict and psychological distress (Conger et al., 2010), and disrupted parenting that put children at risk of long-term psychological problems (Masarik and Conger, 2017). Patterns of non-resident father contact may also be an important mechanism underlying differential effects of biological father absence on depressive symptoms. Evidence suggests that early life family dissolution is associated with a gradual decline in the frequency of contact and involvement between the non-resident father and the child over time (Chase-Lansdale et al., 1995; Hawkins et al., 2006; Koster et al., 2021; Meggiolaro and Ongaro, 2015). Arguably, fathers who leave when the child is older had more opportunities to become involved in child rearing during the early years, and involved fathers are more likely to remain in contact with their children (De Graaf and Fokkema, 2007; Westphal et al., 2014), reducing the risk of adverse mental health in offspring of separated parents (Flouri, 2006).

There was also evidence of sex specific effects of biological father absence on trajectories of offspring depressive symptoms. Specifically, females whose fathers were absent in early childhood had higher depressive symptoms trajectories throughout adolescence compared to their father present counterparts, but this difference had narrowed by age 24. These findings are in line with previous research emphasising adverse effects of parental divorce (Chase-Lansdale et al., 1995) and biological father absence on offspring depressive symptoms, particularly for females (Culpin et al., 2013). Similar patterns of depressive symptoms trajectories in females from divorced compared to nondivorced families have been previously reported (Ge et al., 2006; Hankin, 2009; Stapinski et al., 2013). The rise in female depressive symptoms during early to middle adolescence may coincide with early pubertal transition, particularly in the context of biological father absence (Culpin et al., 2014), and a range of other developmental and psychosocial stressors and transitions that may explain increased risk of emotional distress (Ellis, 2004; Mendle et al., 2007). There is strong evidence for an association between father absence and early timing of menarche in girls (Culpin et al., 2014), but it is unclear whether father absence affects pubertal timing in boys (Belsky et al., 2007). The decline in trajectories during transition to young adulthood in females who experienced early childhood biological father absence is noteworthy and requires further research to understand underlying mechanisms. It has been previously suggested that increased levels of autonomy and decreased reliance on parental support (Lapsley and Woodbury, 2016), relational interdependency and formation of intimate pair bonds (Gutman and Sameroff, 2004), which are characteristic of this developmental period, as well as availability of wider support networks (Gorrese and Ruggieri, 2012), may decrease risk for psychological distress in females.

A different pattern of depressive symptoms trajectories was observed for males whose fathers were absent in early childhood. Specifically, these males had similar trajectories to their father present counterparts until early adulthood where early father absent males experienced a steep rise in depressive symptoms between the ages of 22–24. Vulnerabilities such as renewed sense of parental loss and lack of appropriate male role models or models of long-term intimate adult relationships (Chase-Lansdale et al., 1995) at the time when the importance of interpersonal relationships becomes more salient may be the underlying exploratory mechanisms.

Females whose fathers were absent later in childhood had higher depressive symptoms trajectories across middle adolescence and into young adulthood compared to their father present counterpart, with the widest gap around ages 16–20 years, which could be a key developmental period to intervene. This finding is consistent with our full sample analyses suggesting that negative effects of father absence later in childhood are stronger for females compared to males. In contrast, males whose fathers were absent later in childhood had the lowest trajectories of all, although these were non-discriminant from their father present counterparts. Based on existing literature, it is possible that that rising trajectories for girls may be in line with the overall pattern of higher lifetime trajectories for females than for males. Still, it is unclear why only males and not females show declining trajectories in response to later childhood father absence. It may be that older children have more mature cognitive and emotional resources to cope with the changing nature of father-child relationship (Kelly, 2003), while sex differences in experiencing and coping with interpersonal emotional distress (Seiffge-Krenke and Stemmler, 2002) could explain the more adverse effect of biological father absence for females than males. There is also some evidence that fathers spend more time with their sons than daughters during marriage (Kalmijn, 2015), a pattern that may persist regarding initiation, frequency and quality of contact with the non-resident fathers following family dissolution (Cheadle et al., 2010; Manning and Smock, 1999). Although inconsistent, evidence suggests that not only sons are more likely to be in contact with their non-resident fathers more than daughters, they also perceive the quality of the relationship to be better (De Wit et al., 2014; Kelly, 2000).

### 4.2 Strengths and limitations

The main strengths are the population-based design with prospectively collected data and repeated measures of depressive symptoms and biological father absence, which reduces the possibility of selection and recall bias. The availability of these data enabled us to model differential effects of biological father absence at distinct periods in childhood on clinical diagnosis of depression and depressive symptoms in early adulthood, as well as longitudinal trajectories of mental health from childhood to early adulthood. Furthermore, the advantage of multiple assessments of depressive symptoms over time (from late childhood to adolescence, across adolescence, adolescence to young adulthood) enabled us to examine at which ages father absence may exert its most prominent effect on offspring trajectories of depressive symptoms and whether the effects were short lived or persistent across development. The availability of rich covariable information enabled us to control for a number of social and contextual confounding factors preceding father departure, including parental conflict, which is an important risk factor for both family dissolution (Kelly, 2000) and adverse offspring mental health (Repetti et al., 2002). Lack of adjustment for preceding parental conflict has been argued as a limitation of existing research on the effect of biological father absence (Amato and Keith, 1991). Although we adjusted for a range of confounding factors to minimise the possibility of confounding bias, we cannot fully eliminate the possibility of unmeasured and residual confounding. Another limitation relates to our measure of biological father absence, which does not capture variations and patterns of non-resident fathers contact and involvement with their children due to the lack of data necessarily to contextualise these heterogenous group of non-resident fathers (Poole et al., 2016). We were also unable to account for the frequency of contact and quality of the relationship between the non-resident father and the child, as well as presence of an alternative father figure. It should be noted that our study is limited by the use of maternal reports of biological father absence and lack of data on fathers' own perceptions of their contact and involvement with their non-resident children (Gibson-Davis, 2008).

The limitation of our study also relates to sample attrition, which is similar of that observed in other population-based studies (Boyd et al., 2013; Fraser et al., 2012). Attrition may undermine validity of our findings, given that participants from lower socio-economic background with higher prevalence of father absence and depression were somewhat under-represented in our study. We addressed bias associated with selection attrition by controlling for a range of factors known to predict missingness and by imputing missing data in outcome and confounders. The pattern of missing data and imputed analyses suggested that attrition may have led to the underestimation of main effects in complete case analyses. Another potential limitation is longitudinal measurement invariance as explored in previous research (Kwong et al., 2021), however, given that depression trajectories are based on a summary score and we are not focusing on specific components or constructs of depression, this impact is likely to be limited.

### 4.3 Conclusions and implications of the research

Our findings suggest that the adverse effects of early childhood biological father absence on offspring mental health may persist across adolescence and into early adulthood. Examining sex and timing effects of father absence on offspring depression at distinct developmental periods, as well as longitudinal trajectories across the life course provides a more nuanced understanding of how these effects change with age and their potential differential impact by sex. Such insights are important for targeted interventions. Further research is needed to examine whether these associations are causal to strengthen present findings, as well as efforts to provide insights into mechanisms underlying sex and timing effects of father absence on depressive symptoms to facilitate development of targeted interventions.

## Funding statement

The UK Medical Research Council and Wellcome (Grant ref.: 217065/Z/19/Z) and the 10.13039/501100000883University of Bristol provide core support for ALSPAC. This publication is the work of the authors and will serve as guarantors for the contents of this paper. A comprehensive list of grants funding is available on the ALSPAC website (http://www.bristol.ac.uk/alspac/external/documents/grant-acknowledgements.pdf).

This research was funded in whole by the Wellcome Trust Research Fellowship in Humanities and Social Science (Grant ref.: 212664/Z/18/Z) awarded to Dr. Culpin. For the purpose of Open Access, the author has applied a CC BY public copyright licence to any Author Accepted Manuscript version arising from this submission. Dr. Pearson was supported by the European Research Commission Grant (Grant ref.: 758813 MHINT). Dr. Kwong is supported by an Economic Social Research Council Postdoctoral Fellowship (Grant ref.: ES/V011650/1) and the 10.13039/100010269Wellcome Trust (Grant ref.: 220875/Z/20/Z). This study was also supported by the 10.13039/501100012295NIHR Biomedical Research Centre at the University Hospitals Bristol and Weston NHS Foundation Trust and the University of Bristol. This publication is the work of the authors who will serve as guarantors for the contents of this paper. The views expressed in this publication are those of the author(s) and not necessarily those of the NHS, the National Institute for Health Research.

## Contributions

IC, HH and ASFK performed statistical analyses. JH provided statistical advice. All authors contributed to writing the manuscript and have approved the final version. IC and ASFK had full access to all of the data in the study and take responsibility for the integrity of the data and the accuracy of the data analysis. All authors report no conflicts of interest.

## Role of the funding source

The funder had no role in the design and conduct of the study; collection, management, analysis, and interpretation of the data; and preparation, review, or approval of the manuscript.

The UK Medical Research Council and Wellcome (Grant ref.: 217065/Z/19/Z) and the University of Bristol provide core support for ALSPAC. This publication is the work of the authors and will serve as guarantors for the contents of this paper. A comprehensive list of grants funding is available on the ALSPAC website (http://www.bristol.ac.uk/alspac/external/documents/grant-acknowledgements.pdf).

This research was funded in whole by the Wellcome Trust Research Fellowship in Humanities and Social Science (Grant ref.: 212664/Z/18/Z) awarded to IC. For the purpose of Open Access, the author has applied a CC BY public copyright licence to any Author Accepted Manuscript version arising from this submission. RMP was supported by the European Research Commission Grant (Grant ref.: 758813 MHINT). ASFK was supported by an Economic Social Research Council Postdoctoral Fellowship (Grant ref.: ES/V011650/1) and the Wellcome Trust (Grant ref.: 220875/Z/20/Z). This study was also supported by the NIHR Biomedical Research Centre at the University Hospitals Bristol and Weston NHS Foundation Trust and the University of Bristol. This publication is the work of the authors who will serve as guarantors for the contents of this paper. The views expressed in this publication are those of the author(s) and not necessarily those of the NHS, the National Institute for Health Research.

## Ethical standards

Informed consent for the use of data collected via questionnaires and clinics was obtained from participants following the recommendations of the ALSPAC Ethics and Law Committee at the time. The authors assert that all procedures contributing to this work comply with the ethical standards of the relevant national and institutional committees on human experimentation and with the Helsinki Declaration of 1975, as revised in 2008.

## Appendix A Supplementary content

Supplementary content Image 1
